# Macromycete Edible Fungi as a Functional Poultry Feed Additive: Influence on Health, Welfare, Eggs, and Meat Quality—Review

**DOI:** 10.3390/molecules30153241

**Published:** 2025-08-01

**Authors:** Damian Duda, Klaudia Jaszcza, Emilia Bernaś

**Affiliations:** 1 Department of Animal Physiology and Endocrinology, Faculty of Animal Science, University of Agriculture in Krakow, Mickiewicza 24/28, 30-059 Krakow, Poland; damian.duda@student.urk.edu.pl (D.D.); klaudia.jaszcza@urk.edu.pl (K.J.); 2 Department of Plant Products Technology and Nutrition Hygiene, Faculty of Food Technology, University of Agriculture in Krakow, 122 Balicka, 30-149 Krakow, Poland

**Keywords:** mushrooms, poultry, feed, nutrition, meat and egg quality

## Abstract

Over the years, macromycete fungi have been used as a source of food, part of religious rites and rituals, and as a medicinal remedy. Species with strong health-promoting potential include *Hericium erinaceus*, *Cordyceps militaris*, *Ganoderma lucidum*, *Pleurotus ostreatus*, *Flammulina velutipes*, and *Inonotus obliquus*. These species contain many bioactive compounds, including β-glucans, endo- and exogenous amino acids, polyphenols, terpenoids, sterols, B vitamins, minerals, and lovastatin. The level of some biologically active substances is species-specific, e.g., hericenones and erinacines, which have neuroprotective properties, and supporting the production of nerve growth factor in the brain for *Hericium erinaceus*. Due to their high health-promoting potential, mushrooms and substances isolated from them have found applications in livestock nutrition, improving their welfare and productivity. This phenomenon may be of particular importance in the nutrition of laying hens and broiler chickens, where an increase in pathogen resistance to antibiotics has been observed in recent years. *Gallus gallus domesticus* is a key farm animal for meat and egg production, so the search for new compounds to support bird health is important for food safety. Studies conducted to date indicate that feed supplementation with mushrooms has a beneficial effect on, among other things, bird weight gain; bone mineralisation; and meat and egg quality, including the lipid profile and protein content and shell thickness, and promotes the development of beneficial microbiota, thereby increasing immunity.

## 1. Introduction

According to data from the Food and Agriculture Organization (FAO), the domestic chicken (*Gallus gallus domesticus*) is the most widespread domesticated animal on Earth. In 2020, its population exceeded 33 billion individuals, of which about 46% were found in Asia. Archaeological data and the current range of birds of the genus *Gallus* indicate that their place of origin is the Indian subcontinent, Southeast Asia, and southern China [1,2,3,4].

Domestic chicken is an animal with a wide range of uses. From the perspective of the food industry, domestic chicken breeds can be divided into laying, dual-purpose, and broilers, with fast-growing broilers being the most common for meat production. Laying hens are usually characterized by low body weight and high egg production efficiency, with a satisfactory egg size, as exemplified by the Leghorn breed. Among dual-purpose chickens, the most popular breeds are those characterized by high egg production and satisfactory slaughter value, e.g., the Black Australorp. Meat chickens are characterized by high slaughter value, high feed conversion, and rapid growth-fattening. When comparing the dual-purpose type with the laying type, males of the former can be successfully used in the meat industry as an alternative to broilers [3,5,6,7,8,9].

Due to its reproductive capabilities, the domestic chicken, apart from being used for food, is also a model animal used in scientific research. Most studies focus on genetic and nutritional aspects. Research in the field of food toxicology mainly concerns the assessment of the impact of feed additives rich in health-promoting ingredients (fruit and vegetable pomace, herbs, bee products, and mushrooms) on feed utilisation, muscle mass gain, and animal immunity [10,11,12,13]. The composition of feed also determines the quality of animal products, such as eggs and meat [14]. These aspects are crucial, especially from the perspective of large-scale farms, which, due to the European Union’s agricultural policy and internal regulations of member states, must limit the use of, among other things, antibiotics that have been standardly used to increase immunity and weight gain [15]. Reducing the excessive use of antibiotics and replacing them with natural raw materials rich in bioactive substances, such as antimicrobial peptides or probiotics, may be an opportunity to slow down the rapidly advancing antimicrobial resistance of microorganisms. This phenomenon is also of great importance for humans, as some of the agents used to treat animals are also used to treat humans [16].

**Figure 1:**
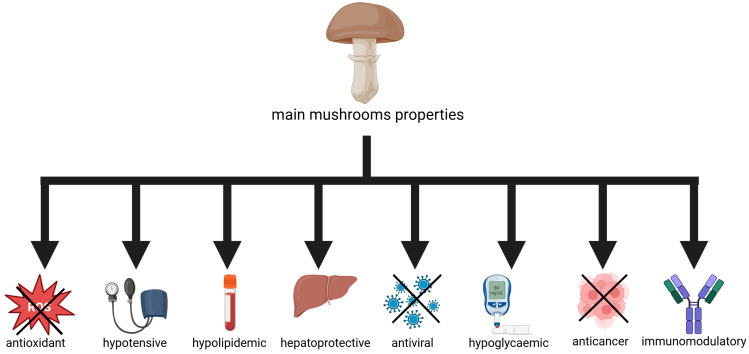
Main functional properties of mushrooms in relation to human medicine.

One of the latest trends in functional feed additives is the use of edible mushrooms as a source of nutraceuticals. Mushrooms are rich in polyphenolic compounds; terpenoids; sesquiterpenes; lactones; sterols; 5-nucleotides; B vitamins; vitamin C; β-glucans; glycoproteins; and macro- and microelements, such as potassium and iron. Due to their wealth of health-promoting ingredients, they are used as immune stimulants and have antiviral, hepatoprotective, hypocholesterolemic, and anticancer effects and improve intestinal peristalsis [13,17] (Figure 1). Some of the most popular species with medicinal properties are Japanese shiitake (*Lentinula edodes*), lion’s mane mushroom (*Hericium erinaceus*), chaga mushroom (*Inonotus obliquus*), Reishi (*Ganoderma lucidum*), and mushrooms of the *Cordyceps* genus [18]. It is not only the case that mushrooms classified as medicinal have a beneficial effect on human health, as this is also the case for species that are frequently consumed, such as the common button mushroom (*Agaricus bisporus*), oyster mushroom (*Pleurotus ostreatus*), and winter mushroom (*Flammulina velutipes*), and such effects have been confirmed by scientific research [19].

## 2. Characteristics and Application of Selected Mushroom Species in the Nutrition of Laying Hens and Broiler Chickens

Macromycete fungi are a source of many bioactive compounds. In addition, they are characterised by a low sugar and fat content and, consequently, a low glycaemic index, which allows them to be classified as functional foods [20]. Thanks to their unique chemical composition and high nutritional value, introducing fungi into the daily diet of both humans and animals can have a beneficial effect on their bodies (Table 1a,b).

### 2.1. Lion’s Mane Mushroom (Hericium Erinaceus)

**Figure 2:**
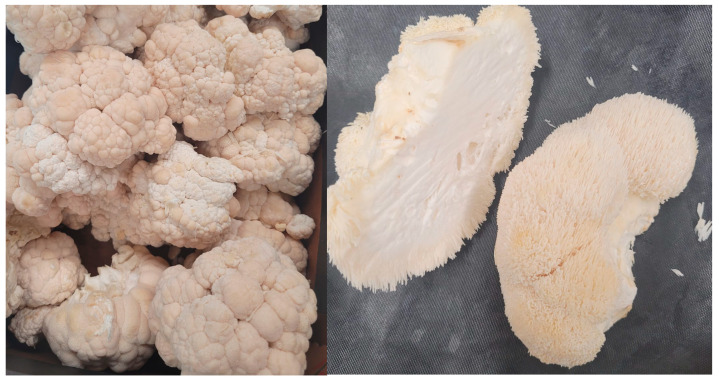
Fruiting bodies of the *Hericium erinaceus*—two different strains (own photo).

Lion’s mane is an edible mushroom belonging to the family *Hericiaceae* and the phylum *Basidiomycota*, whose representatives are found in Europe, Asia, and North America. *H. erinaceus* is a saprophyte that prefers deciduous trees, such as oak, colonising both dead and living specimens [21,22] (Figure 2). This mushroom owes its popularity to traditional Chinese medicine, where it was used as a health-promoting agent for the digestive system [23].

The first documented data on the use of lion’s mane mushrooms come from the 16th-century Chinese pharmacopoeia ‘Compendium of Materia Medica’ [24]. Current research on this species focuses mainly on the evaluation of neuroprotective, neuroregenerative, and neurotrophic properties resulting from the presence of hericenones in the fruiting bodies and erinacine in the mycelium [25,26,27]. In vivo and in vitro tests have shown that 4-chloro-3,5-dimethoxybenzoic methyl ester and erinacine A stimulate nerve growth factor (NGF) synthesis and nerve regeneration in a Sprague–Dawley rat model and may be an important therapeutic element in alleviating the effects of neurodegenerative diseases, such as Parkinson’s and Alzheimer’s disease. A key aspect of the effectiveness of these compounds may be the fact that they easily cross the blood–brain barrier [28].

In the case of poultry, due to the specificity of the test material, studies on lion’s mane mushroom mainly focus on analysing the effects of this mushroom species on the functioning of their reproductive, digestive, and lymphatic systems. Female domestic chickens aged 58 weeks are characterised by very low egg production, which is most often the result of intensive egg production. During the production cycle, hens are significantly exposed to oxidative stress, which is likely to lead to a deterioration in the health of the birds, accelerate the ageing process, and cause a significant reduction in productivity [29,30].

**Table 1:** (**a**) The effects of adding mushrooms to feed on productive performance parameters. (**b**) The poultry health-effect of adding mushrooms to feed.

(a)		
Effect	Research Model	Type of Supplement and Feeding Time
Greater egg production	Hens	HEP from *Hericium erinaceus* (0.5 g/kg, 12 weeks) [30]
Greater weight gain	Broilers	Extract from *Cordyceps sinensis* (0.6 g/kg) with probiotics (6 g/kg, 42 days) [31] FPCM from *Cordyceps militaris* (1–4 g/kg feed, 42 days) [32] SMS from *Flammulina velutipes* (0.5–2.0 g/kg feed, 35 days) [33] Dried *Ganoderma lucidum* (2 g/kg feed, 21 days) [34] Dried *Pleurotus ostreatus* (0.3 g/kg feed, 35 days) [35]
Better feed utilization	Broilers	Extract from *Cordyceps sinensis* (0.6 g/kg) with probiotics (6 g/kg, 42 days) [31] SMS from *Flammulina velutipes* (0.5–2.0 g/kg feed, 35 days) [33] *Agaricus bisporus* stalks (100–150 g/kg feed, 56 days) [36]
Better muscle growth	Broilers	Extract from *Cordyceps sinensis* (0.6 g/kg) with probiotics (6 g/kg, 42 days) [31] FPCM from *Cordyceps sinensis* (1–4 g/kg feed, 42 days) [32] Dried *Agaricus bisporus* (20 g/kg feed, 60 days) [37]
Increased meat quality	Broilers	SMS from *Flammulina velutipes* (0.5–2.0 g/kg feed, 35 days) [33] Dried *Pleurotus ostreatus* (0.3 g/kg feed, 35 days) [35]
Increased egg quality	Hens	FVS from *Flammulina velutipes* (20–40 g/kg feed, 27 weeks) [38] Dried *Agaricus bisporus* (20 g/kg feed, 60 days) [37] *Agaricus bisporus* stalks (100–150 g/kg feed, 56 days) [36]
Increased strength of the eggshell	Hens	FVS from *Flammulina velutipes* (20–40 g/kg feed, 27 weeks) [38]
**(b)**		
**Effect**	**Research Model**	**Type of Supplement and Feeding Time**
Beneficial effect on the immune system	Broilers Chickens Hens Broilers Broilers Chickens	Dried *Ganoderma lucidum* (2 g/kg feed, 32 days) [39] IOFP from *Inonotus obliquus* (8 g/kg feed, 35 days) [40] Extract from *Lentinula edodes* (0.02–0.03 g/kg feed, 21 days) [41] Dried *Pleurotus ostreatus* (0.3 g/kg feed, 35 days) [35] FSW from *Flammulina velutipes* (20 g/kg feed, 42 days) [42] Polysaccharides extract from *Cordyceps militaris* (CMP40, CMP50, 42 days) [43]
Beneficial effect on the intestinal and digestive function	Broilers Hens Broilers	Dried *Ganoderma lucidum* (2 g/kg feed, 32 days) [39] Extract from *Lentinula edodes* (0.02–0.03 g/kg feed, 21 days) [41] Dried *Pleurotus ostreatus* (0.3 g/kg feed, 35 days) [35]
Increases the number of beneficial bacteria	Broilers Hens Broilers Broilers Hens Broilers	Dried *Ganoderma lucidum* (2 g/kg feed, 21 days) [34] Extract from *Lentinula edodes* (0.02–0.03 g/kg feed, 21 days) [41] Dried *Pleurotus ostreatus* (0.3 g/kg feed, 35 days) [35] Dried *Agaricus bisporus* (10–20 g/kg feed, 42 days) [44] Dried *Agaricus bisporus* (10–20 g/kg feed, 60 days) [37] SMS from *Flammulina velutipes* (0.5–2.0 g/kg feed, 35 days) [33]
Increases the height of intestinal villi	Broilers	Dried *Ganoderma lucidum* (2 g/kg feed, 21 days) [34]
Better blood lipid profile	Broilers	HFC from *Hericium erinaceus* (6, 1.2, 1.8 g/kg, 42 days) [45] Dried *Pleurotus ostreatus* (0.3 g/kg feed, 35 days) [35]
Reduction in the cholesterol content in body	Broilers Hens Broilers	HFC from *Hericium erinaceus* (6, 1.2, 1.8 g/kg, 42 days) [45] FVS from *Flammulina velutipes* (20–40 g/kg feed, 27 weeks) [38] SMS from *Flammulina velutipes* (0.5–2.0 g/kg feed, 35 days) [33]
Reduced expression of the HMGR gene	Broilers	HFC from *Hericium erinaceus* (6, 1.2, 1.8 g/kg, 42 days) [45]
Maintenance of hormonal homeostasis and fat metabolism as well as liver and ovarian function	Hens	HEP from *Hericium erinaceus* (0.5 g/kg, 12 weeks) [30]
Less stress	Broilers	Dried *Pleurotus ostreatus* (0.3 g/kg feed, 35 days) [35]
Better antioxidant function	Broilers Hens Hens	Dried *Agaricus bisporus* (20 g/kg feed, 42 days) [46] Dried *Agaricus bisporus* (10–20 g/kg feed, 60 days) [37] ABSR from *Agaricus bisporus* (20–100 g/kg feed, 56 days) [36]
Higher levels of antibodies against Newcastle disease and infectious bursal disease	Broilers Chickens	FSW from *Flammulina velutipes* (20 g/kg feed, 42 days) [42] Polysaccharides extract from *Cordyceps militaris* (CMP40, CMP50, 42 days) [43]
Reduction in ovarian follicle atresia	Hens	FVS from *Flammulina velutipes* (20–40 g/kg feed, 27 weeks) [47]

In addition, liver dysfunction that occurs during this time can lead to physiological disorders, including impaired micronutrient and lipid metabolism, deterioration of endocrine signalling pathways, glycogenolysis, and detoxification processes [48]. In turn, ovarian dysfunction in most birds may have more serious health consequences than in mammals, because due to the asymmetry of ovarian development, manifested by the dominance and full maturity of only the left ovary, ovarian follicle atresia may occur, which in turn determines hormonal destabilisation of the organism [49,50]. As demonstrated by Wu et al. [30], supplementation of feed intended for laying hens with polysaccharides (HEPs) isolated from *H. erinaceus* at a dose of 500 mg/kg for 12 weeks had a beneficial effect on hens’ egg production, as well as on the maintenance of hormonal homeostasis, fat metabolism, and liver and ovarian function. The increase in egg production was probably due to the synthesis of yolk precursors and the development of follicles and ovulation processes as a result of improved ovarian and liver function.

Cholesterol is a key component of animal cell membranes and a precursor of many steroid hormones, bile acids, and vitamin D [51]. However, an excess of this compound, especially in the form of low-density lipoprotein (LDL), can lead to elevated blood cholesterol levels, which is one of the main risk factors for the development of cardiovascular diseases, such as hypertension and atherosclerosis [52,53,54].

In the context of animal production, particularly in intensive systems, an improper feeding system can contribute to the disability or death of fast-growing animals, which in turn generates economic losses [55,56]. Research conducted by Shang et al. [45] showed that enrichment of feed used in broiler chicken nutrition with a 0.6%, 1.2%, and 1.8% fermented *H. erinaceus* mushroom concentrate (HFC) effectively reduced the cholesterol content in chicken muscles and liver, improved their blood lipid profile, and increased SCFA (short-chain fatty acid) production in the caecum and bile acid excretion. The cited authors observed a reduction in the expression of the *HMGR* gene responsible for cholesterol synthesis and an increase in cholesterol metabolism efficiency, which may be a promising method for producing meat with a reduced cholesterol content.

### 2.2. Genus Cordyceps

The fungi of the genus *Cordyceps* are among the most numerous representatives of the family *Clavicipitaceae*, classified in the phylum *Ascomycota*. Approximately 750 species have been described to date, making them one of the most diverse groups within this family. Their natural range includes Europe, Asia, and North America. A characteristic feature of representatives of this genus is their ability to live as parasites, using insects, other arthropods, and fungi as a source of food [57]. These fungi are well-known in traditional Chinese medicine, where they have long been used to treat digestive and urogenital disorders and cancer [58]. The health-promoting properties of *Cordyceps* are mainly due to β-glucans, ergosterol, cordycepin, anthraquinones, naphthoquinones, bioxanthracenes, cytochalazines, alkaloids, polyketides, phenols, flavonoids, pyridines, terpenes, dihydrobenzofurans, and cyclic peptides [59]. Thanks to cordycepin, adenosine, and polysaccharides, *Cordyceps sinensis* boosts immunity by activating immune receptors (TLR4 and DC-SIGN) and signalling pathways (MAPK and ERK1/2), while inhibiting NF-κB. These processes enhance the activity of macrophages and natural killer (NK) cells; promote T regulatory (Treg)/Th2 responses; and increase cytokine levels, including that of IL-6, IL-12, and IFN-γ. Meanwhile, cordycepin reduces inflammation by blocking Akt, NF-κB, and MAPK, thereby lowering the levels of nitric oxide, TNF-α, COX-2, and other pro-inflammatory molecules. Polysaccharides protect against oxidative stress and regulate immunity. Other compounds, such as cordycynins and cordymin, further reduce inflammation and oxidative damage, helping to combat infection, inflammation, and cancer [58].

When it comes to poultry, two species are of particular interest to scientists: *Cordyceps militaris* and *Cordyceps sinensis*. As is widely known, commercial broiler chicken farming is carried out in an intensive system, based on birds selected for efficient feed conversion and rapid muscle growth. Broiler chickens are typically fattened for 5–7 weeks, so there is a high risk of bone and muscle defects (myopathies), the causes of which can be considered in terms of genetics and nutrition [8,60,61,62]. As demonstrated by Khalid Shihab and Hkmat Nafea [31], the use of *C. sinensis* extract at a dose of 600 mg/kg of feed in combination with probiotics (6 g/kg) as a component of complete feed for broiler chickens for a period of 6 weeks resulted in an increase in chicken weight by approximately 8%, compared to a control group. The cited authors suggest that the simultaneous addition of a probiotic and a prebiotic may have contributed to a faster adaptation and stabilisation of the intestinal microbiota, which most likely led to an increase in feed utilisation by the chickens and, consequently, more intensive muscle growth [32,63]. In turn, Han et al. [32] demonstrated that the addition of *C. militaris* fermentation products (FPCMs) to feed intended for broiler chickens has a beneficial effect on bone mineralization, depending on concentration. According to the cited authors, the most beneficial dose was 1 g of FPCM/kg of feed, as it improved the efficiency of feed intake by the chickens and, consequently, increased body weight gain. A higher level of these compounds (4 g/kg) had a negative effect on bone strength and mineral content at later stages of growth, despite increased feed intake and body weight. At the same time, it should also be noted that this study did not show a significant effect of FPCMs on bird mortality. Based on these studies, it can therefore be assumed that the addition of FPCMs from *C. militaris* may be particularly beneficial in the early stages of chicken rearing.

An integral part of commercial poultry farming is the emergence of diseases, the main etiological factors of which may be viruses, bacteria, fungi, and parasites [64]. One of the most dangerous viral diseases is Newcastle disease, caused by highly virulent strains of *Avian paramyxoviruses* 1 (APMV-1), also known as NDV. When symptoms appear, making a correct diagnosis is very difficult, as the symptoms are similar to those of avian influenza, infectious bronchitis, fowl cholera, mycoplasmosis, infectious laryngotracheitis, and psittacosis [65]. Infection with *A. paramyxoviruses* most often occurs as a result of contact between poultry and wild birds and their droppings [64]. Therefore, vaccination is crucial [66]. Wang et al. [43] evaluated the effect of polysaccharides extracted from *C. militaris* (CMP40 and CMP50) on improving the efficacy of a Newcastle disease vaccine in chickens. The results suggest that both polysaccharides significantly stimulated lymphocyte proliferation, both in vitro and in vivo, with CMP40 showing a stronger effect. The best therapeutic effects were observed with low doses of CMP40 (2 mg/mL) and high doses of CMP50 (8 mg/mL). In addition, both polysaccharides increased serum antibody levels, as confirmed by ELISA and HI tests. Furthermore, CMP40 and CMP50 increased the concentration of INF-γ and IL-4 cytokines, enhancing both the cellular and humoral responses of the body. The results obtained suggest that polysaccharides from *C. militaris* may be effective natural adjuvants that enhance the efficacy of vaccines against Newcastle disease.

### 2.3. Winter Mushroom (Flammulina velutipes)

*F. velutipes* is an edible mushroom belonging to the *Basidiomycota* phylum, *Agaricales* order, and *Physalacriaceae* family, found in Europe, Asia, North America, and Australia [67]. It is commercially known as enoki or winter mushroom and is one of the most popular and highly valued edible mushrooms in the world. Due to its anticancer, antioxidant, immunomodulatory, hypotensive, hypolipidemic, and hypoglycaemic properties, this mushroom has found applications in traditional Chinese medicine, mainly in the treatment of liver, stomach, and intestinal diseases [68,69]. In addition to the fruiting bodies, spent mushroom substrate (SMS), the residue from their cultivation, which is rich in protein, carbohydrates, and vitamins, also has health-promoting properties. As demonstrated by Srinual et al. [33], supplementation of feed used in the nutrition of ROSS 308 broiler chickens with SMS (0.5–2.0 g/kg of feed) contributed to an increase in feed intake efficiency and, consequently improved their growth rate while reducing stomach and wing weight and increasing skeletal weight. In addition, a reduction in total blood cholesterol by 4% and its LDL fraction by 20% was shown. The feeding regime had no effect on meat quality, including pH values, colour (both breast and thigh), drip loss, cooking loss, and shear force. Furthermore, there were beneficial changes in the broilers’ intestinal microflora, manifested by an increase in beneficial microorganisms (*Lactobacillus* sp.) and a reduction in the number of pathogens (*E. coli* and *Salmonella* sp.). Enriching the feed with SMS had a positive effect on intestinal function, reducing crypt depth and increasing the height of intestinal villi. The results suggest that SMS can successfully replace antibiotics in animal nutrition, which have a negative impact on the microflora and the functioning of the digestive and immune systems, particularly in young organisms [70].

According to Liu et al. [71] and Arulnathan et al. [72], the production capacity of laying hens decreases due to ovarian ageing processes combined with age-related hormonal changes, which in turn affects egg quality. A study on the use of stem waste (FVS) derived from the cultivation of *F. velutipes* in the nutrition of laying hens (Hy-Line Brown) over a period of 27 weeks was conducted by Sun et al. [38]. As a result of their experiment, the authors showed that a 4% FVS feed supplementation was most beneficial, as it reduced ovarian follicle atresia and increased steroid hormone levels and the expression of FSH (follicle-stimulating hormone) and LH (luteinising hormone) receptors, which promotes follicle maturation. The feeding method used did not affect the egg yolk weight, egg index, egg yolk colour, eggshell thickness, protein height, and Haugh unit but increased egg protein mass and strengthened the eggshell. In addition, a reduction in blood cholesterol levels and body fat in hens was demonstrated. The best results were obtained with a dose of 4%. In turn, Mahfuz et al. [42], studying the effect of enriching the feed of Arbor Acres broiler chickens with *F. velutipes* stem waste (FSW), noted that a 2% addition of FSW resulted in higher levels of antibodies against Newcastle disease and infectious bursal disease, compared to those fed standard feed and with the addition of the antibiotic. Furthermore, the addition of FSW increased the levels of immunoglobulin IgG and cytokines (interleukin-2, interleukin-4, and interleukin-6) in serum, thereby contributing to increased immune resistance in broilers. The likely cause of the increased immunity was the presence of beta-glucans, known for their immunomodulatory properties [73]. As for the serum metabolic profile, no significant changes were found in most of the parameters studied.

### 2.4. Chaga (Inonotus obliquus)

*I. obliquus*, better known to society as Chaga, is a parasitic fungus of the *Hymenochaetaceae* family found in Europe, Asia, and North America, living primarily on birch trunks [74]. Historical data indicate the use of Chaga in folk medicine since ancient times. Historical chronicles suggest that the use of Chaga in the 12th century helped remove a cancerous tumour from a Kiev prince [75]. *I. obliquus* can be consumed in the form of infusions or concentrates, which have a positive effect on health [76]. The observation of a decrease in cancer incidence as a result of consuming infusions among the population of Siberian gulags in the Soviet Union led to the recognition of the therapeutic properties of Chaga by the Ministry of Health in 1955 [47]. This species is credited with anticancer, antioxidant, antiviral, antibacterial, anti-inflammatory, immunomodulatory, hypoglycaemic, hypolipidemic, hepatoprotective, anti-diabetic, anti-obesity, hepatoprotective, renoprotective, and anti-fatigue properties. *I. obliquus* owes its properties to bioactive compounds belonging to, among others, polyphenols, polysaccharides, triterpenoids, sterols, and melanins [77,78]. Currently, Chaga is valued for its rich content due to its ability to modulate the immune response by stimulating cytokine production, strengthening the body’s defences against oxidation, and inhibiting inflammation. In cosmetics, Chaga helps protect against UV radiation, brightens the skin, and counteracts ageing thanks to its melanin, betulinic acid, and tyrosinase-inhibiting compounds [79]. An interesting bioactive compound with medicinal properties is oxysterol inotodiol, isolated from Chaga. Studies in mice have shown that inotodiol exerts its therapeutic properties mainly through potent inhibition of mast cell activation, which plays a key role in acute sepsis. The anti-sepsis effect of inotodiol alleviates clinical symptoms without significantly affecting the levels of common pro-inflammatory cytokines, such as IL-6, TNF-α, and IL-1β, suggesting a mechanism of action that goes beyond direct cytokine inhibition [79].

Most studies on this species are concerned with its use in human medicine, while for poultry nutrition, the focus is on studying its effect on the animal immune system. Zhang et al. [40] showed that *I. obliquus* fermentation products (IOFPs) administered to chickens at a dose of 0.8% of diet for 7 days before twice vaccination with NDV LaSota live vaccine can enhance the cellular and humoral immune response of chickens vaccinated against Newcastle disease virus. *Inonotus obliquus* exerts its immunostimulatory effect mainly through polysaccharides, peptides, triterpenoids, lanosterol, and amino acids, which strengthen immune functions by stimulating T-cell proliferation, increasing the population of CD4^+^ and CD8^+^ T cells and stimulating the production of cytokines, such as IFN-γ and IL-4.

### 2.5. Ganoderma lucidum

*G. lucidum* is a basidiomycete fungus widely used in medicine, found in the subtropical and temperate climates of Asia, Europe, Africa, North America, and South America [80]. *G. lucidum* exerts its healing properties primarily due to its polysaccharides and triterpenes, which modulate the immune system by activating macrophages, lymphocytes, and natural killer cells and stimulating cytokine production. It also has strong anti-inflammatory and antioxidant properties and supports liver protection. In addition to its medicinal properties, this mushroom is used for food purposes, especially in China, Korea, and Japan. Different nomenclatures are used in different geographical areas for this species: Lingzhi in China, Reishi in Japan, Youngzhi in Korea, and Linh chi in Vietnam. *G. lucidum* has been used in Chinese medicine for over 2000 years. Its medicinal properties were first described in Shen Nong Ben Cao Jing, the earliest Chinese pharmacopoeia (25–220 CE). At that time, it was noted that it alleviates cough, asthma, anxiety, insomnia, palpitations, pulmonary insufficiency, shortness of breath, and loss of appetite [81]. The potential medicinal value of *G. lucidum* was unknown to Western civilisation until the 20th century [82]. Current studies indicate that *G. lucidum* may help maintain longevity and vitality, and its industrial value is estimated at over USD 2.5 billion [83]. The latest scientific and clinical studies have shown that *G. lucidum* contains polysaccharides, triterpenoids, steroids, sterols, nucleotides, fatty acids, and other biologically active substances [84]. A number of studies have confirmed that *G. lucidum* has anticancer [85], antioxidant [86], anti-inflammatory [87], anti-diabetic [88], and immunomodulatory [89] activities. In recent years, this species has gained interest among scientists in relation to poultry farming. Research indicates that broiler feed supplementation with 0.2% dried fruiting bodies of *G. lucidum* results in greater and faster weight gain in animals and improves intestinal and immune system function. In addition, feed intake was lower in the mushroom-supplemented groups, indicating better utilisation efficiency of individual nutrients [39]. The results of this study also indicate that supplementation with *G. lucidum* increased the number of lactic acid bacteria in the hens’ intestines, as well as the height of intestinal villi. Changes in the gut microbiota improve digestion and nutrient breakdown by producing enzymes and short-chain fatty acids that nourish intestinal cells. An increased villus height, in turn, increases the intestinal absorptive surface area, allowing for more efficient nutrient absorption [34].

### 2.6. Agaricus bisporus: Button Mushroom

*Agaricus bisporus*, commonly known as the button mushroom, is an edible mushroom species belonging to the *Agaricaceae* family, with a long history of cultivation dating back to the early 18th century [90]. This species is valued in cuisine for its unique flavour, which is largely due to 5′-nucleotides and amino acids that create the Umami flavour (MSG flavour) [91]. Mushroom fruiting bodies, like those of other mushroom species, are a good source of nutrients and bioactive compounds, such as dietary fibre, proteins, exo- and endogenous amino acids, polysaccharides, lectins, phenolic compounds, indole compounds, lovastain, ergothioneine, ergosterol, ergocalciferol, B vitamins, and micro- and macroelements [92]. The bioactive compounds contained in mushrooms have anticancer [93], antibacterial [94], anti-inflammatory [95], antioxidant [96], antiviral [97], and hepatoprotective potentials [98]. Poly- and oligosaccharides from these mushrooms have been used as immune enhancers and have been shown to possess antibacterial, antiviral, and antiparasitic activities in chicken. Moreover, the introduction of fermentable polysaccharides from fungi into poultry feed can support *Lactobacillus* spp. and *Bifidobacterium* spp. populations in the intestines.

With regard to poultry, few scientific studies have mainly focused on assessing the impact of these mushrooms on the functioning of the digestive system, the composition of the intestinal microbiota, and the productive capacity (body weight of broiler chickens and egg production) of the animals. A study on the effect of feeding feed enriched with *A. bisporus* fruiting bodies on intestinal morphology and microflora composition of broiler chickens was described by Giannenas et al. [44]. The authors showed that one-day-old female broiler chickens fed for 42 days with feed enriched with dried mushrooms at 10 and 20 g/kg had a better composition of intestinal microbiota, and a significant increase in the number of *Lactobacillus* spp. and *Bifidobacterium* spp. in the caecum and *Lactobacillus spp*. in the ileum was recorded, compared to control animals. In their subsequent study, Giannenas et al. [46] showed that the above feeding regime had a beneficial effect on muscle mass and antioxidant status of tissues, with an optimal dose of 20 g/kg of feed. In turn, Han et al. [37], studying the effect of feeding laying hens for 60 days with feed supplemented with 10–20% *A. bisporus* stalks, demonstrated a beneficial change in the composition of the intestinal microbiota of the animals, manifested by an increase in the number of beneficial *Alloprevotella* bacteria, especially at a 10% supplementation. With regard to egg quality, a significant increase in the Haugh unit from 72 to 84–86 was observed, and with a 15 and 20% mushroom addition, an improvement in yolk colour from 7.1 to 7.5–7.7 was observed. In addition, the antioxidant function of the organism and protein metabolism improved. Furthermore, studies have shown that in order to replace soybean meal with mushrooms, their share would have to be 10–15%. In turn, Yang et al. [36] showed that enrichment of laying hens’ feed with dried *A. bisporus* stem residue (ABSR) of 2 to 10%, regardless of the percentage in the feed, did not significantly affect egg production, egg quality (chamber height, albumen height, Haugh units, and thick albumen), and preservation effect during storage for 3 weeks at 25 °C. However, the authors noted that this feeding regime improved feed utilisation by the hens and increased the content of antioxidants in blood serum and egg yolk. The most beneficial was a 5% addition of mushrooms to the feed.

### 2.7. Oyster Mushroom (Pleurotus ostreatus)

*Pleurotus* spp., especially *Pleurotus ostreatus,* commonly known as ‘oyster mushrooms’, are one of the most popularly cultivated mushroom species in Europe, Africa, and Asian countries (India, South Korea, China, Taiwan, Japan, and Vietnam) [99]. *P. ostreatus* was first cultivated in the USA in 1900 [100]. The fruiting bodies of this species are highly valued as food not only for their flavour but also for their nutritional and health-promoting properties [101,102]. *P. ostreatus* is rich in dietary fibre, including polysaccharides and oligosaccharides, proteins, vitamins (riboflavin and niacin), minerals (potassium and selenium), and lovastatin [103]. Literature data indicate that this species can also be used in poultry nutrition. Poly- and oligosaccharides present in the cell walls of fungi are particularly important in this case, because they can have a positive influence on the microorganisms in the intestines, thereby promoting growth and acting as prebiotics. Research conducted by Bormon et al. [35] indicates that supplementation of broiler chickens’ feed with dried fruiting bodies of *P. ostreatus* at a dose of 300 mg/kg has a beneficial effect on body weight gain, meat quality, and the functioning of the immune system and digestive tract of birds, compared to a control group and a group receiving an antibiotic (chlortetracycline). The authors reported an increase in blood haemoglobin levels from approx. 9.5 g/dL to 12.2 g/dL and erythrocyte counts from approx. 2.4 × 10^6^/μL to 3.1 × 10^6^/μL, while triglyceride levels decreased from approx. 185 mg/dL to 145 mg/dL and total cholesterol from approx. 185 mg/dL to 150 mg/dL. In addition, the heterophile-to-lymphocyte ratio decreased, indicating improved immunity and lower stress levels in the birds. In the case of the intestinal microbiota, the number of pathogenic bacteria—*E. coli* and *Salmonella* spp.—decreased from 7.58 to 6.23 and from 7.53 to 6.22 log10 cfu/g, respectively, while the number of beneficial bacteria—*Lactobacillus* spp.—increased from 7.21 to 7.60 log10 cfu/g. The studies also suggest that supplementation with *P. ostreatus* at a dose of 300 mg/kg is safe, as higher concentrations of mushrooms may potentially lead to undesirable effects, such as nutrient absorption disorders. In contrast, with regard to other animal species, Adams et al. [104] found that supplementation of piglet feed with 25 g/kg of powdered *P. ostreatus* may lead to nutrient absorption disorders, which may negatively affect the growth and health of animals. Therefore, further research on optimal doses of *P. ostreatus* supplements in the diet of farm animals is recommended to ensure their health and production performance.

### 2.8. Shitake (Lentinula edodes)

**Figure 3:**
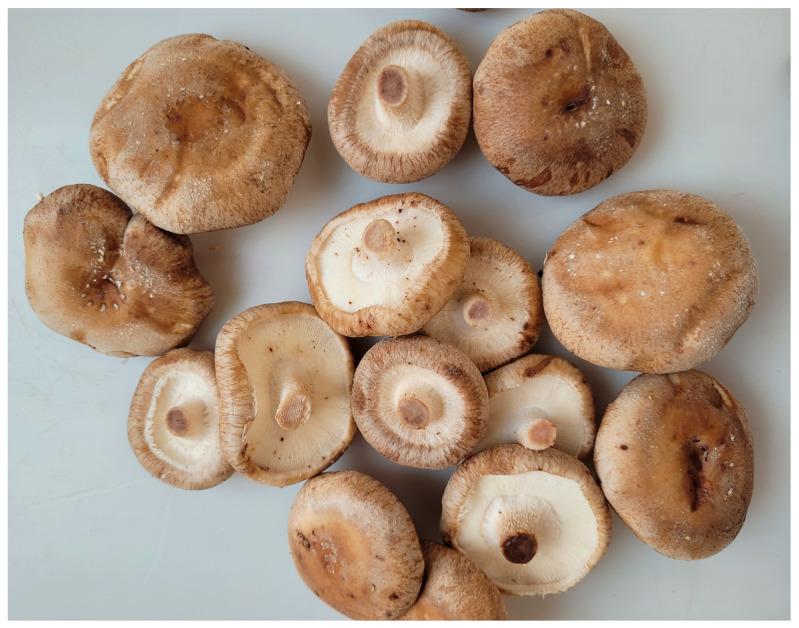
Fruiting bodies of the shiitake mushroom (own photo).

*Lentinula edodes*, also known as shiitake, is a tree-dwelling mushroom that causes the decomposition of dead deciduous tree logs in natural habitats as a result of white rot, mainly from the *Fagaceae* family [105]. *L. edodes* is one of the most commonly consumed and cultivated mushroom species in the world [106]. In 2018, the annual production of *L. edodes* in China exceeded 10,000 million kg, accounting for over 95% of global production [107,108] (Figure 3). Dried shiitake mushrooms have been used in Chinese medicine for centuries to boost immunity, aid digestion, and treat infections, thanks to compounds such as lentinan and eritadenine. Traditionally, they are used for weakness, cough, hypertension, and skin conditions. Shiitake mushrooms support health, thanks to compounds like lentinan (a β-glucan polysaccharide), which activates immune cells (macrophages, T lymphocytes, and NK cells) and stimulates the secretion of cytokines (e.g., IL-1 and TNF-α). Eritadenine, in turn, lowers cholesterol by affecting phospholipid metabolism in the liver, and antioxidant compounds (including ergothioneine) protect cells from oxidative stress and inflammation [109,110].

The fruiting bodies of *L. edodes*, like other mushroom species, are a source of dietary fibre, including glucans, proteins, vitamins (B1, B2, B12, C, D, and E), minerals, terpenoids, and sterols, which have antiviral, antioxidant, anticancer, immunomodulatory, and blood pressure-lowering properties [111,112]. Due to its medicinal properties, *L. edodes* can also be used to improve poultry health and increase production efficiency—increasing egg production from about 88% to 92–93% [113]. Scientific studies indicate a positive effect of *L. edodes* supplementation of laying hens’ feed, especially in terms of improving egg quality and the functioning of the immune and digestive systems [114]. Hwang et al. [113] showed that supplementation of laying hens’ feed with dried *L. edodes* fruiting bodies for 8 weeks at 0.25 and 0.5% improved egg quality, including the Haugh unit, and favourably modified fatty acid composition by increasing linoleic acid as well as total n-6 and polyunsaturated fatty acid contents and reducing the content of palmitoleic acid and α-linolenic acid as well as cholesterol levels (0.5% mushroom supplement). At the same time, no significant effect of mushroom supplementation on other egg quality parameters (egg weight, shape index, shell thickness, protein content, yolk colour, and sensory quality) was observed. In contrast, Guo et al. [41] found that supplementation of feed with *L. edodes* extract can modify the composition of the intestinal microbiota of hens, increasing the number of desirable bacteria from the *Lactobacillus* and *Bifidobacterium* groups and reducing the number of *Bacteroides* spp. and *Escherichia coli*. The obtained results indicate that feed supplementation with *L. edodes* can improve egg quality and proper functioning of the immune and digestive systems, but mushroom concentrations above 0.2 and 0.3% may impair digestion and nutrient absorption by birds.

## 3. Summary

The domestic chicken (*Gallus gallus domesticus*) is the most commonly domesticated animal in the world. Currently, chickens are one of the most important species in the food industry, used for meat and egg production. Intensive farming requires increasingly frequent research into alternative, natural feed additives used to improve bird health and the quality of products obtained from them, especially in the context of reducing the use of antibiotics. One promising area of research is the use of edible and medicinal mushrooms, which contain numerous bioactive compounds (polysaccharides (glucans), terpenes, sterols, lovastatin, etc.) which have, among other things, antioxidant, immunomodulatory, anticancer, anti-inflammatory, antiviral, and anticholesterol properties. Scientific research shows that enriching feed used in poultry nutrition (laying hens and broiler chickens) with mushrooms or extracts obtained from them can improve their production parameters but also support poultry health in a sustainable manner and in line with current global trends. However, in the case of using higher doses, due to possible undesirable effects, it is recommended to adjust the amount of mushrooms in the feed to the animal species each time.

It should be emphasized that mushroom cultivation generates large amounts of spent mushroom substrate (SMS), whose organic matter content ranges from 40 to 60% [115]. Despite the metabolization of some nutrients, this raw material can be reused in a circular economy [116]. The conversion of waste into resources will become increasingly important due to demographic forecasts for the 21st century, which indicate a continuing trend of global population growth, reaching 11.184 billion in 2100 [117]. Population growth combined with rapid urbanization will exacerbate the existing crisis of limited access to natural resources, including the most valuable ones, such as safe drinking water, which will be limited for more than 50% of humanity for more than a month [118]. Freshwater resources suitable for agriculture and the food industry will therefore continue to decline [119]. Resource constraints always have economic consequences, so it is essential to seek new and broader applications for SMS. To date, the potential of SMS in the production of fertilisers, biogas, biofuels, enzymes, bioactive compounds, feed additives, and feed has been reported [120]. Animal production accounts for 77% of land used for food production, while producing only 18% of food [121]. Therefore, in animal production, it should become increasingly important to feed animals with food that is not edible for humans but is edible for them [122]. Macromycete mushrooms are also currently a relatively expensive food product, and their cultivation is complicated. Therefore, from the current perspective, SMS may be the only realistic way to introduce mushrooms into animal feed. However, new feed additives can be placed on the EU market only after obtaining authorisation under the procedure laid out in Regulation (EC) No. 1831/2003. This procedure is based on a scientific assessment of the safety, efficacy, and quality of the additive [123].

## Data Availability

Inquiries can be directed to the corresponding author.
